# Composition, In Vitro Antioxidant and Antimicrobial Activities of Essential Oil and Oleoresins Obtained from Black Cumin Seeds (*Nigella sativa* L.)

**DOI:** 10.1155/2014/918209

**Published:** 2014-02-06

**Authors:** Sunita Singh, S. S. Das, G. Singh, Carola Schuff, Marina P. de Lampasona, César A. N. Catalán

**Affiliations:** ^1^Chemistry Department, DDU Gorakhpur University, Gorakhpur, Uttar Pradesh 273009, India; ^2^INQUINOA-CONICET, Instituto de Química Orgánica, Facultad de Bioquímica Química y Farmacia, Universidad Nacional de Tucumán, T4000INI San Miguel de Tucumán, Argentina

## Abstract

Gas chromatography-mass spectrometry (GC-MS) analysis revealed the major components in black cumin essential oils which were thymoquinone (37.6%) followed by p-cymene (31.2%), **α**-thujene (5.6%), thymohydroquinone (3.4%), and longifolene (2.0%), whereas the oleoresins extracted in different solvents contain linoleic acid as a major component. The antioxidant activity of essential oil and oleoresins was evaluated against linseed oil system at 200 ppm concentration by peroxide value, thiobarbituric acid value, ferric thiocyanate, ferrous ion chelating activity, and 1,1-diphenyl-2-picrylhydrazyl radical scavenging methods. The essential oil and ethyl acetate oleoresin were found to be better than synthetic antioxidants. The total phenol contents (gallic acid equivalents, mg GAE per g) in black cumin essential oil, ethyl acetate, ethanol, and n-hexane oleoresins were calculated as 11.47 ± 0.05, 10.88 ± 0.9, 9.68 ± 0.06, and 8.33 ± 0.01, respectively, by Folin-Ciocalteau method. The essential oil showed up to 90% zone inhibition against *Fusarium moniliforme* in inverted petri plate method. Using agar well diffusion method for evaluating antibacterial activity, the essential oil was found to be highly effective against Gram-positive bacteria.

## 1. Introduction

Preservation of food degradation, mainly by oxidation processes or by microorganism activity, during production, storage, and marketing is an important issue in the food industry. There is currently a large interest in substituting synthetic food preservatives and synthetic antioxidants for substance that can be marketed as natural. Synthetic antioxidants such as gallates, butylated hydroxytoluene (BHT), butylated hydroxyanisole (BHA), and tert-butyl hydroquinone (TBHQ) were the first preservatives designed for widespread industrial use. However, some physical properties of BHA and BHT, such as their high volatility and instability at elevated temperatures, strict legislation on the use of synthetic food additives, and consumer preferences, have shifted the attention of manufacturers from synthetic to natural antioxidant [1]. It is well known that most spices possess a wide range of biological and pharmacological activities.

Black cumin (*Nigella sativa *L.) belonging to family Ranunculaceae is a spice that has been used for decades for both culinary and medicinal purposes. It is also used as a natural remedy for asthma, hypertension, diabetes, inflammation, cough, bronchitis, headache, eczema, fever, dizziness, and influenza [2]. The seeds are known to be carminative, stimulant, and diuretic [3]. The essential oil from the seeds of this herbaceous plant has been found to contain high concentrations of thymoquinone and its related compounds such as thymol and dithymoquinone, which have been implicated in the prevention of inflammation [4], antioxidant activities [5], such as quenching reactive oxygen species, antimicrobial activity [6], and anticarcinogenic and antiulcer activity [2].

The present paper deals with the chemistry and antioxidative and antimicrobial behavior of essential oil and oleoresins (extracted in ethanol, ethyl acetate, and n-hexane) of black cumin seeds.

## 2. Materials and Methods

The seeds of black cumin were purchased from the local market of Gorakhpur, Uttar Pradesh, India. A voucher specimen was deposited at the herbarium of the Faculty of Science, DDU Gorakhpur University.

### 2.1. Reagents

Thiobarbituric acid (TBA), 1,1′-diphenyl-2-picrylhydrazyl radical (DPPH), and linoleic acid are of Acros (New Jersey, USA); butylated hydroxytoluene (BHT), butylated hydroxyanisole (BHA), and propyl gallate (PG) are of S D Fine Chemicals Ltd., Mumbai, India. Folin-Ciocalteu reagent and gallic acid were from Qualigens Chemicals Ltd., Mumbai, India, and Qualikems Chemicals Ltd., New Delhi, India, respectively. Tween 20 and ferrozine were from Merck Pvt. Ltd., Mumbai, India. Ampicillin was purchased from Ranbaxy Fine Chemicals (New Delhi), India. Crude linseed oil was obtained from local oil mill in Gorakhpur. All solvents used were of analytical grade.

### 2.2. Sample Extraction

Powdered seeds of black cumin (250 g) were subjected to hydrodistillation in Clevenger apparatus for 3 h according to the method recommended by European Pharmacopoeia, [7]. A volatile oil with light orange characteristic odour was obtained with yield of 0.9%. It was dried over anhydrous sodium sulphate and the sample was stored at 4°C before use.

Oleoresins were obtained by extracting 30 g of powdered spice with 300 mL of various solvents (ethanol, ethyl acetate, and n-hexane) for 3 h in Soxhlet extractor. Evaporation of the solvents at reduced pressure gave viscous extracts. The oleoresins were stored in freezer until further use.

### 2.3. Chemical Characterization

#### 2.3.1. Gas Chromatography-Mass Spectrometry (GC-MS)

Analysis of the volatile oils and oleoresins was run on a Hewlett Packard (6890) GC-Ms system coupled to a quadruple mass spectrometer (model HP 5973) with a capillary column of HP-5MS (5% phenyl methylsiloxane; length = 30 m, inner diameter = 0.25 mm, and film thickness = 0.25 *μ*m). GC-MS interphase, ion source, and selective mass detector temperatures were maintained at 280°C, 230°C, and 150°C, respectively. Carrier gas used was helium with a flow rate of 1.0 mL min^−1^. The oven temperature was programmed as follows.

For essential oil: at 60°C for 1 min then increased from 60 to 185°C at the rate of 1.5°C min^−1^ and held at the rate of 9°C min^−1^ and held at 275°C for 2 min.

For oleoresin: 60°C for zero min then increased from 60 to 300°C at the rate of 1.5°C min^−1^ and held at the rate of 5°C min^−1^ and held at 300°C for 10 min.

### 2.4. Identification of Components

Most of the components were identified on the basis of comparison of their retention indices and mass spectra with published data [6, 8, 9], and computer matching was done with the Wiley 275 and National Institute of Standards Technology libraries provided with the computer controlling GC-MS systems. The retention indices were calculated using a homologous series of n-alkanes C_8_–C_18_ and C_8_–C_22_ for essential oil and oleoresins, respectively, which are reported in Tables 1 and 2.

## 3. Antioxidant Activity

The antioxidant activity is system dependent and according to the method adopted and lipid system used as substrate. Hence, different methods have been adopted in order to assess antioxidative potential of black cumin oil and its oleoresins are as follows.

### 3.1. Chelating Activity on Ferrous Ions

The chelating activity of the aqueous and ethanolic extract on ferrous ions (Fe^2+^) was measured according to the method described by Decker and Welch [10]. Aliquots of 1 mL of different concentrations of the samples were mixed with 3.7 mL of deionized water. The mixture was incubated with FeCl_2_ (2 mM, 0.1 mL). After incubation the reaction was initiated by addition of ferrozine (5 mM and 0.2 mL) for 10 min at room temperature, and then the absorbance was measured at 562 nm in a spectrophotometer. A lower absorbance indicates a higher chelating power. The chelating activity of the extract on Fe^2+^ was compared with that of EDTA that was used as positive control. Chelating activity was calculated using the following formula:

(1) Chelating  activity(%)  =[1−(Absorbance  of  sampleAbsorbance  of  control)]×100.

### 3.2. Scavenging Effect on DPPH

The DPPH assay constitutes a quick and low cost method, which has frequently been used for the evaluation of the antioxidative potential of various natural products, [11]. Due to its odd electron, DPPH gives a strong absorption band at 517 nm (deep violet colour). In the presence of a free radical scavenger, this electron becomes paired, resulting in the absorption loss and consecutive stoichiometric decolorization with respect to the number of electron acquired. The absorbance change produced by this reaction is assessed to evaluate the antioxidant potential of the test sample. 5, 10, 15, and 20 *μ*L of the sample were added to 5 mL of 0.004% methanol solution of DPPH. After a 30 min incubation period at room temperature, the absorbance was read against a blank at 515 nm. All determination was performed in triplicate and results were performed in triplicate and results are reported as scavenging effect (%) versus concentration in Figure 2.

### 3.3. Estimation of Total Phenolic Content (TPC)

TPC were determined using the Folin-Ciocalteu reagent method described by Singleton and Rossi [12]. Gallic acid stock solution (1000 *μ*g mL^−1^) was prepared by dissolving 100 mg of gallic acid in 100 mL of ethanol. Various dilutions of standard gallic acid were prepared from this stock solution. Calibration curve (Figure 3) was plotted by mixing 1 mL aliquots of 10–100 *μ*g mL^−1^ of gallic acid solutions with 5.0 mL of Folin-Ciocalteu reagent (diluted tenfold) and 4.0 mL of sodium carbonate solution (75 g L^−1^). The absorbance was measured after 30 min at 20°C at 765 nm.

## 4. Evaluation of Antioxidant Activity for Linseed Oil System

For present investigation, crude linseed oil, having initial peroxide value 5.2 meq kg^−1^, was taken to assess the antioxidant activity of black cumin oil and its oleoresins. This oil is most frequently used edible oil in central Europe and is rather unstable because of the presence of substantial amount of linoleic acid. The antioxidant activity of volatile oil and extract was examined by comparing the activity of known antioxidants such as PG, BHT, and BHA by the following peroxide value and thiobarbituric acid value methods.

### 4.1. Peroxide Value Method

For measuring the peroxide value (PV), a modified oven test was used [13]. The antioxidant activity of black cumin oil and its oleoresins in different solvents were compared with the synthetic antioxidants, such as PG, BHT, and BHA. For this purpose, calculated quantities of each (200 ppm) were dissolved to 30 g of linseed oil in an open mouthed beaker. The mixtures were thoroughly homogenized and placed in incubator at 80°C. The peroxide values (meq of oxygen kg^−1^) were measured in every seven days and test was replicated for three times. A control sample was prepared under similar condition without any additive. The effects of oil and oleoresins in term of peroxidation at 90°C are shown in Figure 4.

### 4.2. Thiobarbituric Acid Value (TBA)

TBA value of different samples was determined according to the method previously reported [13]. About 100 mg of oil sample was dissolved in 25 mL of 1-butanol. A 25 mL aliquot of the above solution was mixed thoroughly with 5.0 mL of TBA reagent (200 mg TBA in 100 mL of 1-butanol) and incubated at 95°C. After 2 h, the reaction mixture was cooled to room temperature under running water and absorbance was measured at 530 nm with Hitachi-U-2000 spectrophotometer (Tokyo, Japan). At the same time, a reagent blank (without TBA reagent) was also done. The thiobarbituric acid value (meq of malondialdehyde per g) was calculated as

(2) $$\begin{array}{c} \text{TBA value}=\frac{50\times \left(A-B\right)}{M}, \end{array}$$

where *A* is absorbance of the test sample, *B* is absorbance of the reagent blank, and *M* is mass of the sample.

### 4.3. Determination of Antioxidant Activity in Linoleic Acid System

Antioxidant activity of black cumin oil and its oleoresins was compared to synthetic standards according to the ferric thiocyanate method in linoleic acid emulsion [14]. The reaction medium contained black cumin oil and oleoresins at the concentration of 1 mg/100 mL of absolute ethanol (2 mL), an emulsion of 2.51% linoleic acid in ethanol (2 mL), 4 mL of 0.05 M-phosphate buffer (pH = 7.0), and 2 mL of distilled water. The solution (10 mL) was mixed and incubated at 40°C in the dark. The same solution, without any additives, was taken as control sample. At regular intervals during incubation, 0.1 mL aliquot of the mixture was diluted with 9.7 mL of 75% ethanol followed by the addition of 0.2 mL of 30% ammonium thiocyanate and 0.1 mL of 20 mM of FeCl_2_ in 3.5% HCl; the absorbance of red colour was measured at 500 nm in Hitachi-U-2000 spectrophotometer (Tokyo, Japan). The control and standard were subjected to the same procedure except for the control, where there was no addition of sample and for the standard 1 mL of sample were replaced with 1 mg of PG, BHT, and BHA. These steps are repeated every 48 h until the control sample reached its maximum. Low absorbance value indicates the efficiency of the test samples to inhibit lipid oxidation. The results were reported as incubation time versus absorbance in Figure 6.

## 5. Antimicrobial Investigations

### 5.1. Antifungal Assay

The antifungal activity of the essential oil and extract against various pathogenic fungi, *Aspergillusflavus* (1884), *Aspergillus niger* (2479), *Fusarium moniliforme* (1893), *Fusarium graminearum* (2088), and *Penicillium viridicatum* (2007), were tested by the inverted petri plate [15] and poison food medium methods [16]. All the fungi cultures were procured from the Microbial Type Culture Collection (MTCC) and their reference numbers are given in the parentheses. The cultures were maintained in Czapek agar medium. Each test was replicated three times and fungi toxicity was measured in terms of percentage mycelial inhibition calculated with the following equation:

(3) $$\begin{array}{c} \text{mycelial inhibition}\left(\%\right)=\left[\frac{\left(d_{c}-d_{t}\right)}{d_{c}}\right]\times 100, \end{array}$$

where *d*_*c*_ and *d*_*t*_ are the average diameters of the mycelial colony of the control and treated sets, respectively.

### 5.2. Antibacterial Assay

The essential oil and extract were individually tested against a panel of microorganisms using agar well diffusion method [17]. Three Gram-positive bacteria, *Staphylococcus aureus *(3103), *Bacillus cereus *(430), and *Bacillus subtilis *(1790), and two Gram negative bacteria, *Escherichia coli *(1672) and *Pseudomonas aeruginosa *(1942). All the bacterial strains were procured from the Microbial Type Culture Collection (MTCC), Institute of Microbial Technology (Chandigarh, India), and their reference numbers are given in parentheses. The bacterial cultures were grown on nutrient agar medium and stored at 4°C. In order to prepare a bacterial strain for test, initially one loopful of bacterial culture was transferred from slant to nutrient broth solution (10 mL) and was stored at 37°C for 24 h. The control plate without the addition of essential oil or extract containing DMSO was also maintained under the same conditions. After incubating for 24 h at 37°C, all plates were examined for any zones of growth inhibition and the diameters of these zones were measured in millimeters.

## 6. Statistical Analysis

For the essential oil or oleoresin, three samples were prepared for assays of every antioxidant and antimicrobial attribute. The data are presented as mean (standard deviation of three determinations (data are not shown). Statistical analyses were performed using a one-way analysis of variance [18]. A probability value of *P* < 0.05 was considered to be significant.

## 7. Results and Discussions

### 7.1. Phytochemistry

Careful and detailed interpretations of the experimental GC-MS data (EM fragmentation, retention indices) were carried out which permitted identification of a large number of components in essential oil and oleoresins (Tables 1 and 2).  Table 1 shows identification of 33 components in black cumin oil, representing about 90.2% of the total amount. The major component in black cumin oil was thymoquinone (37.6%) followed by p-cymene (31.4%), *α*-thujene (5.6%), thymohydroquinone (3.4%), longifolene (2.0%), and carvacrol (1.4%). Burits and Bucar [8] characterized many components in black cumin essential oil such as thymoquinone (27.8–57.0%), p-cymene (7.1–15.5%), carvacrol (5.8–11.6%), trans-anethole (0.25–2.3%), 4-terpineol (2.0–6.6%), and 1.0–8.0% of longifolene. These results are slight different from the work reported by Hajhashemi et al. [19] who reported that p-cymene (37.3%) and thymoquinone (13.7%) were the major components of black cumin. Singh et al. [6] also reported p-cymene as the major component in the black cumin essential oil.

From Table 2, it is evident that, in ethanol oleoresin, 19 components constitute 96.4% of the total weight; in the ethyl acetate oleoresin, a total of 19 components making 90.9% of the whole mass and, in case of n-hexane oleoresin, 19 compounds constituting about 85.2% of the total weight were identified. The oleoresins were mainly comprised of unsaturated fatty acids and their different esters. The major components in all three oleoresins were linoleic acid (unsaturated fatty acid) followed by glyceryl linoleate, glyceryl palmitate, oleic acid, and other minor components. The presence of unsaturated fatty acids in oleoresins was well supported by the various reported work [20–26].

### 7.2. Antioxidant Investigations

#### 7.2.1. Chelating Activity on Ferrous Ions

**Figure 1:**
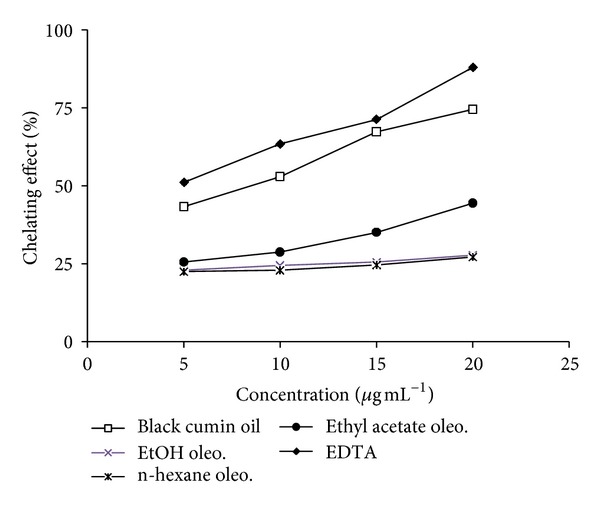
Chelating effect of black cumin oil and its different oleoresins.

The ferrous ion (Fe^2+^) chelating effect of black cumin oil and its different oleoresins is presented in Figure 1. The chelating activity of the extracts was concentration dependent. Black cumin oil exhibited higher chelating activity in comparison to the oleoresins but was not effective chelator as EDTA. Maximum chelating of metal ions at 200 *μ*g mL^−1^ for black cumin oil and EDTA was found to be 74.56% and 87.90%, respectively, whereas the oleoresins were less effective in metal chelation and their metal chelating activity ranges from 22.1 to 44.5%.

#### 7.2.2. Scavenging Effect on DPPH Radical

**Figure 2:**
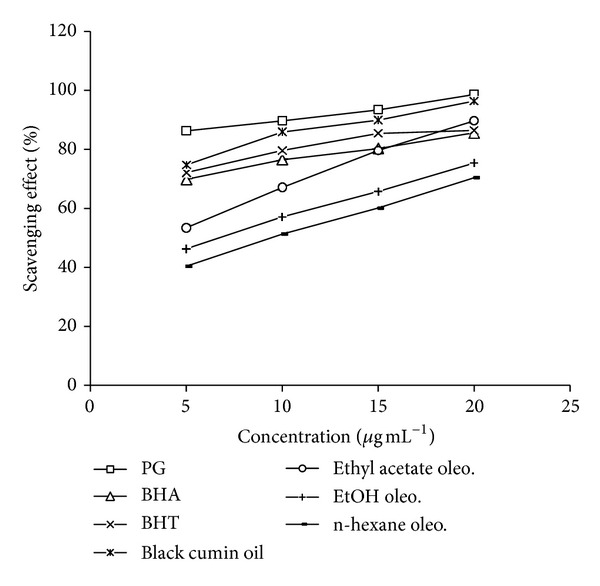
Scavenging effect (%) of black cumin oil and its oleoresins on DPPH radical.

DPPH^•^ is a stable radical showing a maximum absorbance at 515 nm. In DPPH^•^ assay, the antioxidant was able to reduce the stable radical DPPH to the yellow-colored diphenylpicrylhydrazone. The method is based on the reduction of DPPH^•^ in alcoholic solution in the presence of a hydrogen-donating antioxidant due to formation of the nonradical form DPPH-H in the reaction. DPPH^•^ is usually used as a reagent to evaluate free radical and accepts an electron or hydrogen radical to become a stable diamagnetic molecule. The disappearance of the DPPH radical absorption at 515 nm by the action of antioxidants is taken as a measure of antioxidant activity. The scavenging effects of black cumin oil and oleoresins on DPPH radical linearly increased as concentration increased from 5 to 20 *μ*g mL^−1^ (Figure 2). At 20 *μ*g mL^−1^ the scavenging activity of black cumin oil and ethyl acetate oleoresin was 95.4% and 89.75%, respectively, comparatively higher than BHT and BHA but lower than PG. However the scavenging activity of BHA, BHT, and PG was more effective at lower concentration and was 69.8%, 72.1%, and 86.3% at 5 *μ*g mL^−1^ but as the concentration increases the differences in scavenging activity between BHA, BHT, and oleoresins (specially ethyl acetate) become less significant. Ethanol and n-hexane oleoresins showed moderate scavenging activity.

#### 7.2.3. Estimation of TPC

**Figure 3:**
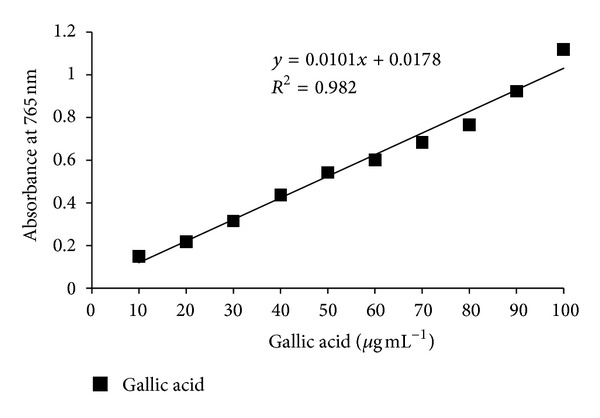
Calibration curve of gallic acid.

The amount of total phenols was determined with Folin-Ciocalteu reagent. Gallic acid was used as standard compound. The absorbance for various dilutions of gallic acid with Folin-Ciocalteu reagent and sodium carbonate was obtained and found standard curve equation: *y* = 0.0101*x* + 0.0178, *R* ^2^ = 0.982 (Figure 3). The total phenol contents (gallic acid equivalents, mg GAE per g) in black cumin essential oil, ethyl acetate, ethanol and n-hexane oleoresins were calculated as 11.47 ± 0.05, 10.88 ± 0.9, 9.68 ± 0.06, and 8.33 ± 0.01, respectively. The value suggests that the black cumin oil and its oleoresins have lesser amount of total phenols [27]. The differences in the total phenolic content among the samples might be due to many differences, such as the environmental conditions, genetic background, or agricultural techniques applied.

### 7.3. Antioxidant Assays in Linseed Oil System

**Figure 4:**
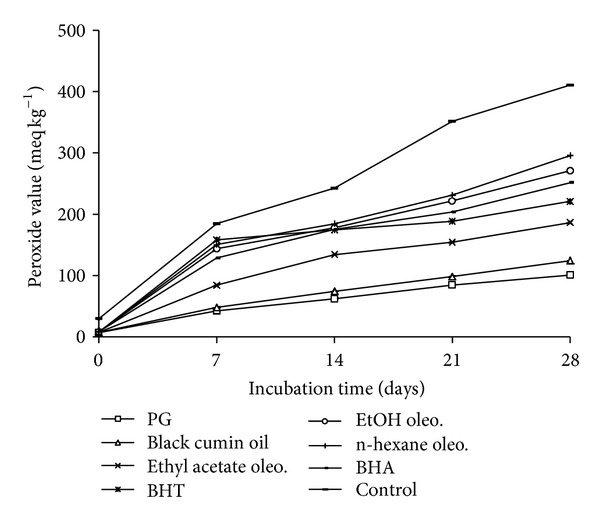
Inhibitory effect of black cumin oil and its oleoresins on the primary oxidation of linseed oil measured using peroxide value method.

The changes of PV in linseed oil of all investigated samples are presented in Figure 4. The rate of oxidative reactions in linseed oil with additives was almost similar to that of the blank sample. The stability of the linseed oil samples to the formation of peroxides can be ranked in the following descending order:

  PG > Black  cumin  oil > Ethyl  acetate  oleo. > BHT > BHA > EtOH  oleo. > n-hexane  oleo. > Control.

**Figure 5:**
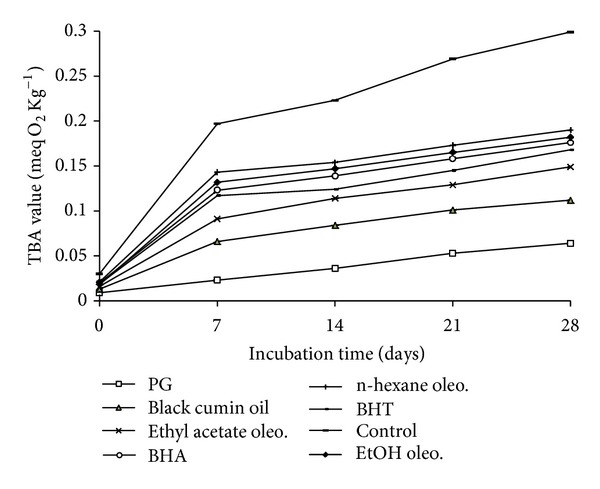
Inhibitory effect of black cumin oil and its oleoresins on the primary oxidation of linseed oil measured using TBA value method.

Simultaneously with the measurements of PV, changes in secondary product such as malondialdehyde, the compound used as an indicator of lipid peroxidation was measured by TBA values (Figure 5), were also determined after every seven days. Black cumin oil and its ethyl acetate oleoresin showed strong inhibition at 0.02% concentration as compared to BHT and BHA (*P* < 0.05) but lower than PG whereas the ethanol and n-hexane oleoresin showed moderate inhibition at 0.02% concentration as compared to the other additives. From the above results, it should be confirmed that formation of primary oxidation species, peroxides, was quite similar with the secondary oxidation products and the changes of both oxidation characteristics are in a good correlation.

### 7.4. Antioxidant Activity in Linoleic Acid System

**Figure 6:**
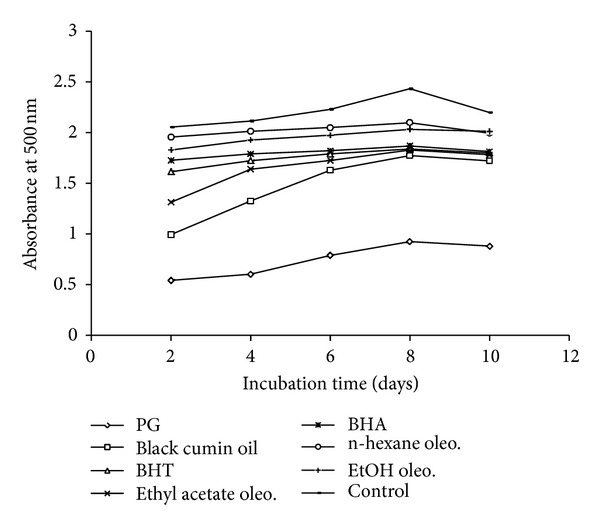
Inhibitory effect of black cumin oil and its oleoresins on the primary oxidation of linoleic acid system measured using ferric thiocyanate method.

The FTC method was used to measure the amount of peroxides at the primary stage of linoleic acid peroxidation (Figure 6). Since the concentration of the peroxide decreases as the antioxidant activity increases, the intensity of the pigment will be reduced, leading to lower absorbance. Absorbance values of the control as well as black cumin oil and its oleoresins increase until day 10 and then decreased on day 12 due to the malondialdehyde formation from linoleic acid oxidation. There was a significant difference (*P* < 0.05) between the control and the tested essential oil and oleoresins. As can be seen in Figure 6, both black cumin oil and oleoresins showed good antioxidative property in the linoleic acid system and were significantly (*P* < 0.05) different from the control.

Antioxidant activities of essential oils and oleoresins may be related to the diverse compounds present in them including terpenes, sesquiterpenes, and phenolic acids, which act in various ways such as inhibition of peroxidation, scavenging the radicals, and chelating the metal ions. The main constituents of black cumin oil were thymoquinone (37.6%) and p-cymene (31.4%) with minor amounts of longifolene, carvacrol, and thymohydroquinone which were responsible for the antioxidant activity of black cumin oil [8, 9, 19, 28–31]. Furthermore, free radical scavenging effects of these components were studied on the reactions generating reactive oxygen species such as superoxide anion radical, hydroxyl radical using spectrophotometric methods [5]. The results obtained using different assays were well correlated with the previous work reported be many workers [5, 30] who found thymoquinone as a main constituent responsible for the activity. It has been suggested that phenolic content is correlated with the antioxidant activity [32]. It is considered that the antioxidant activity of phenolic compounds is due to their high redox potentials, which allow them to act as reducing agents, hydrogen donors. Thymoquinone was also present in small amount with higher percentage of unsaturated fatty acid, linoleic acid, in different oleoresins. Studies [33] have shown that these unsaturated fatty acids have anti-rather than pro-oxidant activity but still research has been going on for the exact role of unsaturated fatty acids against the oxidative stress.

### 7.5. Antimicrobial Investigations

Using inverted petri plate technique (Table 3), the volatile oil exhibited more than 90% zone inhibition for *F. moniliforme* and *P. viridicatum*. It was also found to be highly effective in controlling the growth of *Aspergillus *species and *F. graminearum* where (50%) and (65%) zone inhibition was observed, respectively. For other tested fungi, the essential oil exerted less activity. However using the same method, the oleoresins have revealed less activity except for *F. moniliforme*, in which only up to 40% mycelial zone inhibition was obtained. Moreover using food poison technique (Table 4), the volatile oil showed clear zone of growth inhibition against *F. graminearum* at 10 *μ*L. The volatile oil showed strong antifungal activity against all tested *Aspergillus* species in the food poison method. Ethyl acetate and ethanol oleoresin showed up to 30% zone inhibition at 10 *μ*L dose. The n-hexane oleoresin showed very feeble inhibition zone in both techniques. The lower antimicrobial efficacy of the oleoresins is due to their low volatility [34].

The antibacterial investigations were undertaken using agar well diffusion method (Table 4). Using this method, the black cumin oil has shown better activity than oleoresins and commercial bactericide, that is, ampicillin. The volatile oil was found to be highly effective against *B. subtilis, B. cereus, and S. aureus* and showed complete zone of inhibition at 3000 ppm concentration whereas in oleoresins more than 20 mm inhibition was obtained for Gram-positive bacteria. In addition, more than 20 and 25 mm zone inhibition was obtained for *P. aeruginosa* and *E. coli*. The results obtained using agar well diffusion method were well correlated with the earlier reported work [6, 30, 35] where black cumin seed oil has been shown to be effective against a wide spectrum of organisms, bacteria like *B. cereus, B. subtilis, S. aureus, S. epidermis, E. coli,* and *P. aeruginosa*.

The results obtained in antimicrobial investigations of black cumin oil and oleoresins were in good agreement with the previous reported work [36]. Thymoquinone, p-cymene (monoterpene), longifolene (sesquiterpene), and thymohydroquinone were responsible for strong antimicrobial activity of black cumin oil [29]. El Alfy et al. [35] isolated thymohydroquinone as antimicrobial compound from the volatile oil of *Nigella sativa* seeds. Oleoresins have high concentration of unsaturated fatty acids along with thymohydroquinone in small amount which is responsible for its moderate antimicrobial effects. Long chain fatty acids like linoleic acid and oleic acid were previously reported to possess antibacterial and antifungal activity [36–39]. *p*-Cymene is not an efficient antimicrobial compound when used alone, but it potentiate the activity of compounds like carvacrol [40]. The antimicrobial activity of essential oils can often be correlated to its content of phenolic constituents. The type of bacteria also has an influence on the effectiveness of the volatile oil and oleoresins. Gram-negative bacteria were generally less susceptible than Gram-positive bacteria [41]. The difference in the susceptibility of the bacteria arises as a result of differences in their cell membrane structure which is more complex in case of Gram-negative bacteria. The antimicrobial activity of a given essential oil may depend on only one or two of the major constituents that make up the oil. However, increasing amounts of evidence indicate that the inherent activity of essential oils may not only rely exclusively on the ratio in which the main active constituents are present, but also on interactions between these and minor constituents in the oils and oleoresins.

## 8. Conclusions

Seeds of black cumin seem to possess magical properties and have been worked out extensively. This study revealed that black cumin essential oil and its oleoresins constitute a good alternative source of essential fatty acids compared with common vegetable oil. The present results showed that essential oil and oleoresins of black cumin exhibited higher antioxidant activity than synthetic antioxidants. These findings could be used to prepare multipurpose products for pharmaceutical applications and its usage as dietary source of antioxidant should be considered largely for alleviating and ameliorating diseases.

## Figures and Tables

**Table 1:** Chemical composition of essential oil obtained from black cumin seeds analyzed by GC-MS.

Compounds	%MS	RI^#^	Identification^Φ^
α-Thujene	5.6	919	MS, RI, co-GC
α-Pinene	1.4	928	MS, RI, co-GC
Sabinene	0.8	967	MS, RI, co-GC
β-Pinene	1.7	973	MS, RI, co-GC
*α*-Phellandrene	0.1	1003	MS, RI
*α*-Terpinene	0.2	1012	MS, RI, co-GC
*p*-Cymene	31.4	1019	MS, RI, co-GC
Limonene	1.0	1024	MS, RI, co-GC
1,8-Cineole	0.1	1025	MS, RI, co-GC
*γ*-Terpinene	0.2	1050	MS, RI, co-GC
*trans*-Sabinene hydrate	0.1	1101	MS, RI
Unidentified B*	6.8	1113	−
Terpinen-4-ol	1.0	1172	MS, RI, co-GC
*p*-Cymen-8-ol	Trace	1179	MS, RI
*α*-Terpineol	Trace	1189	MS, RI, co-GC
Cuminal	Trace	1240	MS, RI
Carvone	Trace	1241	MS, RI
Thymoquinone	37.6	1248	MS, RI
*trans*-Sabinene hydrate acetate	0.1	1258	MS, RI
Bornyl acetate	0.2	1285	MS, RI
Thymol	0.2	1289	MS, RI
Carvacrol	1.4	1295	MS, RI
*α*-Longipinene	0.5	1353	MS, RI
Longifolene	2.0	1405	MS, RI
Thymohydroquinone	3.4	1559	MS, RI
10-epi-*γ*-Eudesmol	0.3	1625	MS, RI
*β*-Eudesmol	0.5	1652	MS, RI
*α*-Eudesmol	0.4	1655	MS, RI

Total	90.2%		

Trace < 0.05%; ^#^the retention index was calculated using a homologous series of n-alkanes C8–C18; ^Φ^Co-GC: coinjection with an authentic sample. Percentages were obtained from electronic integration Trace measurements using a selective mass detector.

**Table 2:** Chemical composition of oleoresins obtained from black cumin (*Nigella sativa* L.) seeds in different solvents analysed by GC-MS.

Compounds	M1	M2	M3	RI^#^	Identification^Φ^
*α*-Thujene	Trace	0.4	0.6	919	MS, RI, co-GC
*p*-Cymene	0.9	2.8	2.2	1019	MS, RI, co-GC
Unidentified A	0.5	0.5	0.4	1092	−
Thymoquinone	5.7	6.1	3.7	1248	MS, RI
Carvacrol	0.4	Trace	Trace	1295	MS, RI, co-GC
*α*-Longipinene	Trace	Trace	Trace	1353	MS, RI
Longifolene	0.5	0.6	0.3	1405	MS, RI
Thymohydroquinone	2.5	1.6	0.5	1559	MS, RI
Palmitic acid, ethyl ester	2.8	Trace	Trace	1979	MS, co-GC
Linoleic acid, methyl ester	0.6	0.5	0.5	−	MS, co-GC
Linoleic acid, ethyl ester	11.6	Trace	0.6	−	MS, co-GC
Oleic acid, ethyl ester	4.6	Trace	0.2	−	MS, co-GC
Oleic acid	0.3	Trace	0.2	−	MS, co-GC
Linoleic acid	33.0	43.9	27.7	−	MS,
Linoleic acid, butyl ester	0.9	5.7	16.0	−	MS
Oleic acid, butyl ester	1.2	4.5	7.3	−	MS
Glyceryl palmitate	3.7	1.6	2.3	−	MS
Glyceryl linoleate	27.7	21.9	23.1	−	MS
Sitosterol	Trace	1.3	Trace	−	MS, co-GC

Total	96.4%	90.9%	85.2%		

Trace < 0.05; ^#^the retention index was calculated using a homologous series of n-alkanes C8–C20; ^Φ^Co-GC: coinjection with an authentic sample.

Percentages were obtained from electronic integration measurements using selective mass detector.

M1: ethanol oleoresin; M2: ethyl acetate oleoresin; M3: n-hexane oleoresin.

**Table:** (a)

Mycelial zone inhibition at different doses^a^ of sample (%)						
Samples	Doses (µL)	AN	AF	FM	FG	PV
Black cumin oil	5	43.6 ± 0.30	45.7 ± 1.3	71.2 ± 0.50	39.7 ± 0.14	34.7 ± 0.6
	10	80.9 ± 0.36	70.3 ± 1.8	89.7 ± 0.20	65.7 ± 0.17	87.6 ± 0.7
EtOH oleoresin	5	5.7 ± 0.20	8.9 ± 0.20	17.8 ± 2.4	10.0 ± 0.20	9.9 ± 0.36
	10	11.2 ± 0.30	13.2 ± 0.30	41.9 ± 0.3	11.3 ± 0.14	13.7 ± 0.40
n-Hexane oleoresin	5	0.2 ± 0.44	4.3 ± 0.17	4.5 ± 1.2	2.4 ± 0.36	5.6 ± 0.54
	10	5.5 ± 0.46	7.6 ± 0.14	39.8 ± 0.1	9.1 ± 0.41	9.7 ± 0.6
Ethyl acetate oleoresin	5	19.8 ± 0.20	11.2 ± 0.7	20.1 ± 2.1	19.6 ± 1.1	18.1 ± 0.6
	10	25.2 ± 0.26	16.4 ± 3.6	49.2 ± 2.3	31.1 ± 1.7	20.9 ± 0.8

^a^Average of three replicates.

AN: *Aspergillus  niger*, AF: *Aspergillus  flavus*, FM: *Fusarium  moniliforme*, FG: *Fusarium  graminearum*, and PV: *Penicillium  viridicatum*.

(−): no inhibition.

**Table:** (b)

Mycelial zone inhibition^a^ at different doses of sample (%)						
Samples	Doses (ppm)	AN	AF	FM	FG	PV
Black cumin oil	5	65.9 ± 0.9	60.3 ± 0.1	42.2 ± 1.8	93.2 ± 1.2	50.8 ± 1.5
	10	81.2 ± 1.3	77.8 ± 0.2	61.7 ± 0.7	100 ± 0.8	55.4 ± 0.3
Ethanol oleoresin	5	25.2 ± 2.1	22.4 ± 1.7	−	31 ± 1.9	−
	10	30.3 ± 2.1	29.8 ± 1.4	10.7 ± 0.8	33.7 ± 1.4	−
n-Hexane oleoresin	5	16.7 ± 0.7	14.4 ± 0.6	−	17.9 ± 1.5	−
	10	21.4 ± 2.1	17.4 ± 0.1	−	19.9 ± 2.3	−
Ethyl acetate oleoresin	5	28.4 ± 1.4	23.7 ± 0.4	−	21.4 ± 1.4	−
	10	35.2 ± 1.1	28.4 ± 0.5	15.3 ± 1.2	25.9 ± 0.7	−

^a^Average of three replicates.

AN: *Aspergillus  niger*, *AF*: *Aspergillus  flavus*, FM: *Fusarium  moniliforme*, FG: *Fusarium  graminearum*, and PV: *Penicillium  viridicatum*.

(−): no inhibition.

**Table 4:** Antibacterial activity of black cumin oil and its oleoresinsagainst a few bacterial species using agar well diffusion method.

Diameter of inhibition zone (mm^a^)						
Samples	Doses (ppm^b^)	BS	BC	SA	EC	PA
Black cumin oil	1000	++	25.3 ± 1.4	++	−	18.9 ± 0.17
	3000	++	++	++	20.3 ± 0.14	27.9 ± 0.15
Ethanol oleoresin	1000	16.4 ± 0.81	−	15.6 ± 0.36	−	−
	3000	28.3 ± 0.20	−	25.9 ± 0.42	−	−
n-Hexane oleoresin	1000	11.5 ± 0.31	−	9.1 ± 1.1	−	−
	3000	20.9 ± 1.9	−	13.4 ± 2.6	−	−
Ethyl acetate oleoresin	1000	22.3 ± 0.8	−	17.7 ± 2.3	−	−
	3000	44.3 ± 0.7	−	40.9 ± 1.2	−	−
					
Ampicillin	1000	15.6 ± 0.32	−	9.1 ± 1.1	−	−
	3000	13.2 ± 0.2	−	13.4 ± 2.6	−	−

^a^Average of three replicates; ++ indicates complete inhibition and − indicates no inhibition.

^b^DMSO was used as solvent.

BS: *Bacillus  subtilis*; SA: *Staphylococcus  aureus*; BC: *Bacillus  cereus*. EC: *Escherichia  coli*; PA: *Pseudomonas  aeruginosa*.
